# Noninvasive Quality Assessment of Melt-Grown Cesium Lead Bromide Perovskite by Nuclear Quadrupole Resonance Spectroscopy

**DOI:** 10.1021/acs.chemmater.5c02047

**Published:** 2026-01-08

**Authors:** Lidiia Dubenska, Sebastian Sabisch, Andrii Kanak, Martin Kotyrba, Maksym V. Kovalenko

**Affiliations:** † Department of Chemistry and Applied Biosciences, ETH Zürich, Vladimir-Prelog-Weg 1-5, CH-8093 Zürich, Switzerland; ‡ Empa-Swiss Federal Laboratories for Materials Science and Technology, Überlandstrasse 129, CH-8600 Dübendorf, Switzerland

## Abstract

Melt-grown, highly crystalline CsPbBr_3_ has been intensely investigated as a semiconductor for direct hard radiation detection. While the phase purity and crystallinity of the CsPbBr_3_ ingots are assessed by X-ray diffraction and optical microscopy, the overall quality of the material is ultimately judged by the performance of the final device. The iterative evaluation of crystal quality would greatly benefit from broadening readily accessible structural methods. In this work, we establish nuclear quadrupole resonance (NQR) spectroscopy as a versatile, noninvasive technique for evaluating the quality of melt-grown CsPbBr_3_ ingots. We show that in addition to its inherent utility for probing the local environment around a quadrupolar nucleus, NQR spectroscopy is highly sensitive to crystal orientation and crystallinity, as further supported by ab initio calculations. The key spectroscopic descriptors (linewidth and integrals) can thus be correlated with both macroscopic and microscopic structural features, thereby establishing a robust and rapid method for evaluating crystal quality. Customized resonators can accommodate large ingots and enable measurements directly in the quartz ampule used for melt growth, as well as semiautomated spatial mapping of spectroscopic features across the ingots. For instance, we show that removing the impurities collected near the top of the ingot and subsequent recrystallization improve the homogeneity and overall crystallinity of the samples, highlighting the need for multiple purification steps. We also observe that different crystallographic orientations of crystal domains along the ingot are obtained and preserved in cut crystal disks. These findings pave the way for integrating NQR spectroscopy as a practical, noninvasive tool for in-line or in-situ crystal quality control and guided sample selection.

## Graphical abstract

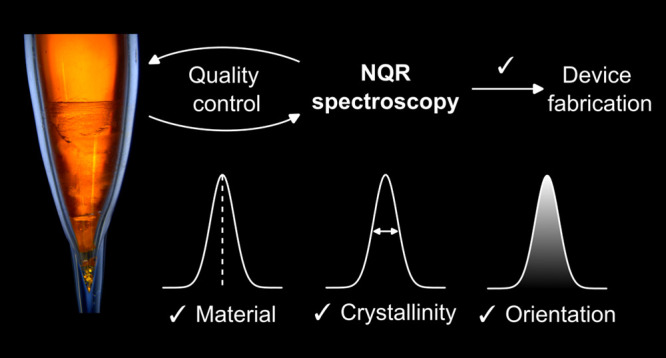

## Introduction

The continued exploration and optimization of materials for hard radiation detectors respond to the pressing needs for affordable, low-dose, and high-spatial resolution medical imaging.^1–3^ Owing to their high-Z composition, lead halide perovskite (LHP) semiconductors are promising active materials for direct hard radiation detection.^4–6^ LHPs benefit from inexpensive and technically facile growth methods^7,8^ and high tolerance of their electronic characteristics to defects and even impurities (they are usually grown from technical or reagent-grade precursors),^9,10^ which contrasts with conventional semiconductors that require ultrahigh purity and crystallinity. The key attributes of their high electronic quality, enabling efficient hard-radiation detection, include resistivity,^2^ short detection times (ns), and large mobility-lifetime products (μτ).^5^ These characteristics are known to substantially deteriorate in samples with reduced crystallinity.^11^ While some LHP compositions using organic cations can be obtained with high crystallinity from solution,^12,13^ CsPbBr_3_ delivers its best electronic performance when grown from melt using the Bridgman-Stockbarger technique.^5,8,14^ All-inorganic CsPbBr_3_ is particularly attractive due to the presence of the nondecomposable and heavy Cs^+^ cation, which enhances high energy attenuation coefficients and enables effective detection with reduced amounts of active material.^15^

Low lattice energy and hence structural softness of LHPs, and their highly entropic character (increased static and dynamic disorder)^16^ comprise a key differentiator from their contenders in the realm of conventional, structurally rigid semiconductors such as Si, CdTe, and GaAs. The nondemanding crystallization of LHPs is somewhat neutralized by the hard-to-track and difficult-to-control microscopic and macroscopic structural disorder and crystal quality. The Bridgman-Stockbarger technique is a standard method for obtaining large, high-quality crystal ingots from their melt.^17,18^ During directional crystallization, impurities present in the material are pushed into the melt and accordingly accumulate at the top of the ingot. Therefore, the top part of the crystal is removed before further recrystallization steps, during which the same directional crystallization from a melt is repeated.

After the initial growth, CsPbBr_3_ undergoes two solid–solid phase transitions at 130 °C (cubic to tetragonal) and 88 °C (tetragonal to orthorhombic) before it reaches the room temperature phase.^19,20^ Lowering of the symmetry oftentimes promotes twin formation, introducing discontinuities in the crystal lattice that reduce charge-carrier mobility and degrade device performance.^21^ Additionally, phase transitions and cooling of the ingot cause changes in the lattice parameters and lead to mechanical stress or even crack formation, particularly in materials with high thermal expansion coefficients, such as those found in CsPbBr_3_.^22^ The micro- and macroscopic structure of such mechanically soft materials may also be altered by standard sample processing, such as cutting and polishing.^23^

The practical utility of a semiconductor crystal as a hard-radiation detector can be fully assessed only after the fabrication and testing of a prototype device. At the heart of this costly, iterative, and tedious exploration and optimization lies material-specific structural characterization, affording, at first, the establishment of the correlation and then a causal link between the crystal quality and detector performance. Optical microscopy can qualitatively evaluate the crystal homogeneity. However, in the case of melt-grown ingots, such analysis is difficult to quantify and is further complicated by the ingot’s spherical geometry. While disk-shaped sections—typically prepared for performance testing—can be assessed with optical microscopy, the technique requires partial destruction of the ingot, and results are not readily comparable, making it unsuitable for standardized quality control. Currently, noninvasive analytical methods for determining crystallinity and crystal orientation within intact ingots are not available. In the context of LHPs, nuclear quadrupole resonance (NQR) spectroscopy of halides has emerged as a powerful and accessible technique to probe the local chemical environment of a nucleus.^20,24–37^ In this work, we propose that NQR spectroscopy can also be effectively applied to assess overall crystal quality in LHPs noninvasively, providing quantifiable quality parameters for evaluation. We conducted the quality assessment of melt-grown CsPbBr_3_ ingots by directly measuring the NQR in the ampule used during the growth. We extract the relationship between spectroscopic descriptors, such as signal width and integral, and the macro- and microscopic characteristics of the sample, showcasing an exceptional sensitivity to the crystallite sizes and the dominating growth direction of the crystallites. Ab initio calculations also corroborate an extensive structural probing range, well beyond the first- and second coordination spheres. By customizing a commercial probe, fast and semiautomated spatial mapping of the crystallinity and crystallographic orientation along the whole ingot is attained, revealing an improved homogeneity and crystallinity of the sample upon subsequent purification and recrystallization. We have also uncovered reorientation in the crystallographic alignment of domains along the ingots. We then show that NQR can guide the cutting of crystal disks with known crystallinity and orientation, enabling noninvasive, guided selection of samples using an approach readily integrated into existing materials-processing workflows.

## Results and Discussion

NQR is a viable technique for probing quadrupolar nuclei exhibiting sufficiently large quadrupolar interactions, such as all the halides (Cl, Br, and I)^26,27^ (Table S1), which have been previously studied in LHPs by NQR spectroscopy.^28–37^ NQR spectroscopy has thus far been mainly used to study phase transitions and mixed ion compositions of LHP powders.^34–36^ A prerequisite to establishing NQR spectroscopy for the quality assessment of LHPs is understanding the origin of the observed resonance. This transition frequency is determined by the quadrupolar interaction—the coupling between the nuclear quadrupole moment (Q) and the intrinsic electric field gradient (EFG) around the nucleus.^24^ For isotopes with a spin of 3/2, such as ^35/37^Cl or ^79/81^Br, only one transition frequency (±1/2 → ± 3/2) is observed in the absence of a magnetic field.

^79^Br has a slightly higher natural abundance than ^81^Br and a larger quadrupole moment (Table S1), leading to a higher resonance frequency, both factors contributing to improved signal-to-noise ratios. Two signals are observed in the NQR spectrum of CsPbBr_3_ at 68.06 and 66.78 MHz (for ^79^Br at room temperature), indicating the presence of at least two inequivalent bromide positions.^37^ CsPbBr_3_ is described in an orthorhombic lattice (space group: *Pbnm* with *a* = 8.25 Å, *b* = 8.21 Å, *c* = 11.76 Å) at room temperature, featuring three inequivalent bromide sites. Two bromides in the crystallographic ab-plane are nearly indistinguishable, as they both form a Pb–Br–Pb bond angle of 157°.^20,38^ We further refer to them as equatorial (Figure 1a). The third bromide, aligned along the *c*-direction, forms a slightly larger angle of 165° with its neighboring Pb atoms, thereby making its environment distinctly different from that of the equatorial bromides.^20,38^ We will refer to this position as axial. The EFG tensor around bromides in CsPbBr_3_, which, together with the quadrupolar moment, determines the observed NQR frequency (S2), was calculated using density functional theory (DFT) to have a prolate shape aligned with the bonds to the neighboring Pb atoms (Figure 1a).^29^ The difference in Pb–Br–Pb bond angles for axial and equatorial positions results in a difference in principal values of the EFG tensor, allowing us to assign the axial bromide to the higher frequency signal (Figure 1b).

**Figure 1:**
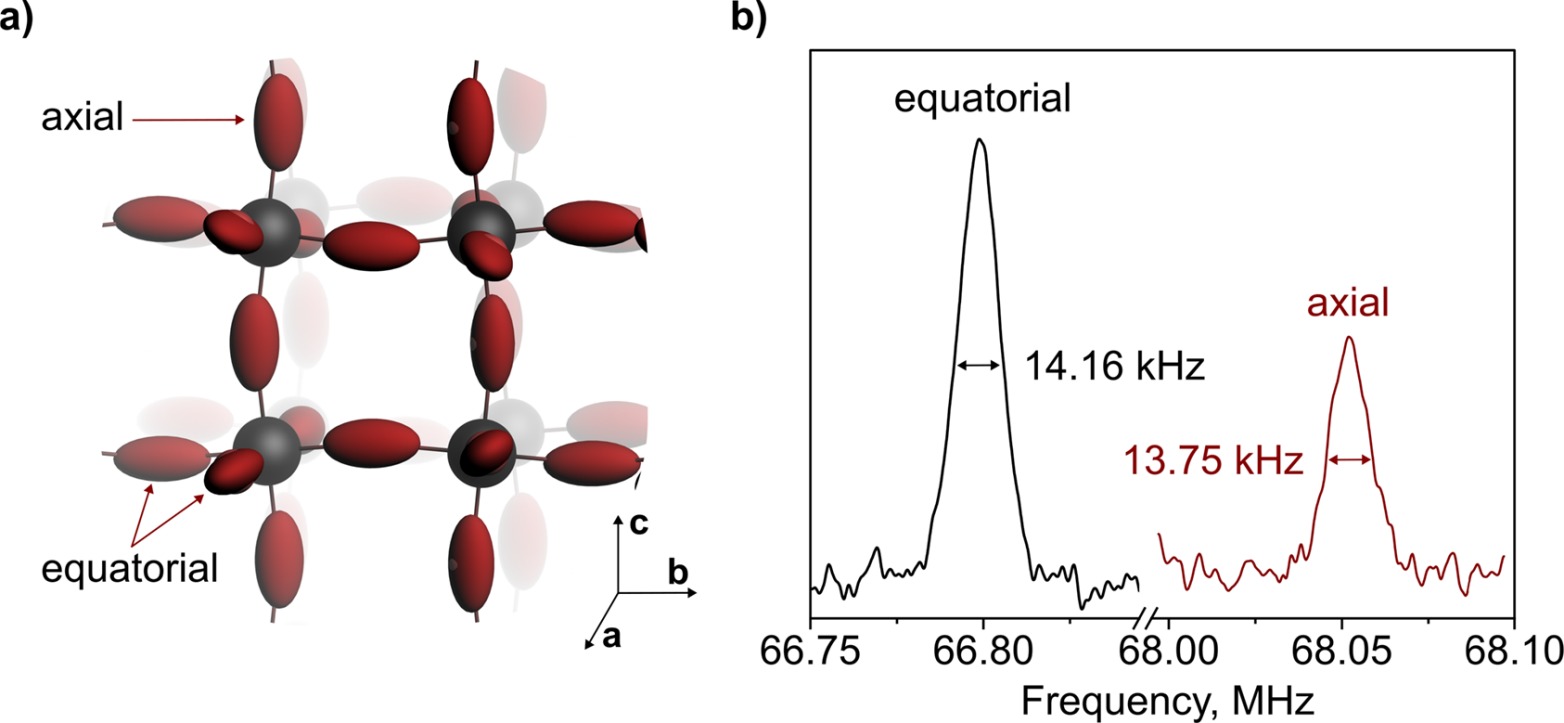
(a) Schematic representation of CsPbBr_3_ with calculated EFG tensors on the ^79^Br nuclei (maroon ellipses); Cs atoms are hidden for clarity. (b) NQR spectrum of CsPbBr_3_ powder at room temperature showing the ^79^Br resonances. Integral ratio of equatorial to axial bromides: 1.89 ± 0.15.

The EFG tensor not only governs the observed NQR frequency but also dictates the alignment of spins, which orient themselves along its largest principal value, V_33_.^39^ In CsPbBr_3_, Pb atoms form bonds with bromides in three orthogonal directions, defining three orthogonal EFG tensors (Figure 1a). As such, the magnetic field (B_1_) generated during the radio frequency (rf) pulse of the NQR experiment will interact with these spins depending on the relative orientation of B_1_ and V_33_. Only those spins which have a component of V_33_ perpendicular to the B_1_ vector can be excited by the rf pulse and will contribute to the signal, giving rise to the most intense NQR signals when V_33_ is fully perpendicular to B_1_.^40^ In a powder, all crystal orientations and, therefore, spin magnetization vectors are equally present. Since the ratio of equatorial to axial bromides is 2:1, the observed NQR signals exhibit an integral ratio of 1.89 ± 0.15 as well (Figure 1b). On the other hand, in highly crystalline materials all EFG tensors of the individual quadrupolar nuclei are aligned, resulting in a dependence of the individual signal integrals on the angle between the respective EFG tensor and the B_1_-vector. This enables the determination of the crystallographic alignment by orientation-dependent measurement of the integral ratio of equatorial to axial bromide intensities. It is worth noting that the NQR signal intensity scales with the square of the resonance frequency when using Faraday induction for detection. Therefore, when signals with significantly different frequencies are compared, a correction should be applied. In this case, however, the frequency difference between equatorial and axial bromides is less than 2%, and we approximate the sensitivity to be equal.

The crystallographic alignment of melt-grown CsPbBr_3_ is hard to determine and predict. First, only Laue X-ray diffractometry is readily available for this task, but it is complicated by the intense twinning. Furthermore, the growth direction from the melt remains unknown. We address this task using NQR by recording the integral ratio of the equatorial to axial signals. This ratio is expected to reveal the crystallographic alignment in CsPbBr_3_ ingots, where three orthogonal EFG tensors define three magnetization vectors (Figure 2a). These vectors form angles α, β, and γ with the applied B_1_ field vector. The signal integral of each spin, according to the Bloch equation,^40^ has a sinusoidal dependence on the reference frame of the applied B1 field (Figure 2b) because only the transverse component of the magnetization vector contributes to the detected signal. Therefore, for a genuine single crystal, the integral ratio *I*_eq_/*I*_ax_ changes according to eq 1:

$$\frac{I_{\mathrm{eq}}}{I_{\mathrm{ax}}}=\frac{\sin(\alpha )+\sin(\beta )}{\sin(\gamma )}$$ 1

Since all three magnetization vectors are orthogonal to one another, not all possible combinations of α, β, and γ exist. They must satisfy the conditions (S3) in eq 2:

$$\cos^{2}\left(\alpha \right)+\cos^{2}\left(\beta \right)+\cos^{2}\left(\gamma \right)=1$$ 2

**Figure 2:**
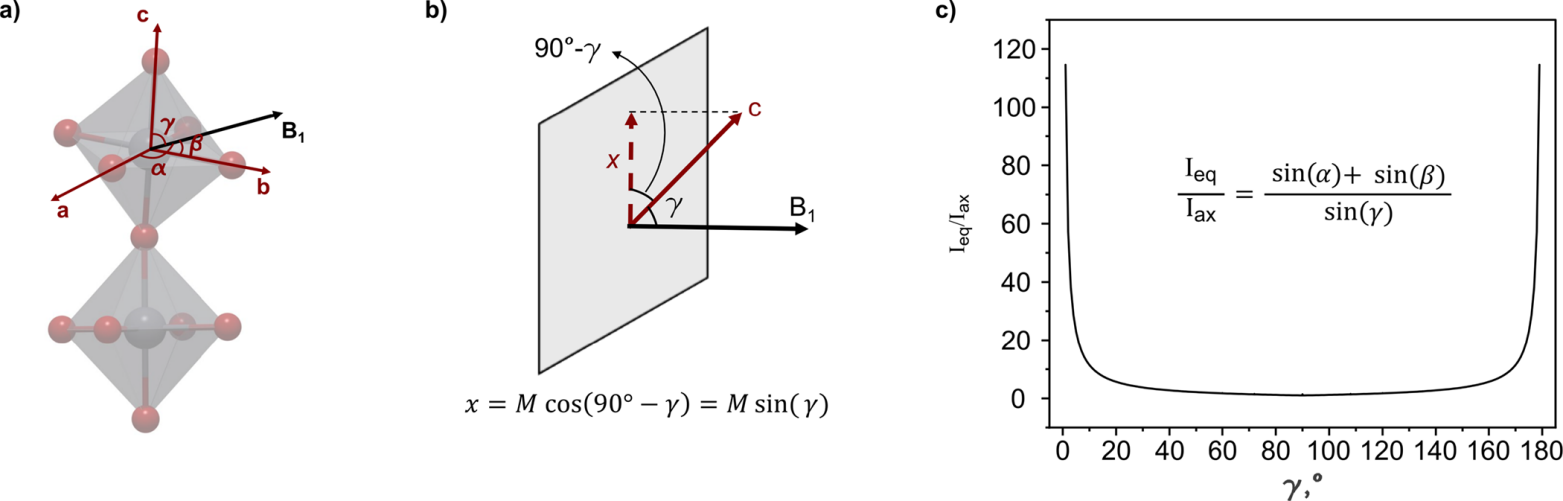
(a) [PbBr_6_] octahedra with three magnetization vectors corresponding to equatorial bromides along *a* and *b* and axial bromides along *c*, which, at any arbitrary crystal alignment, form angles α, β, and γ with the applied B_1_ field vector. (b) Dependence of the observed signal integral of each magnetization vector on its angle to the B_1_ field vector. (c) Integral ratio *I*_eq_/*I*_ax_ profile for an ideal single-domain crystal showing sensitivity to the alignment of the *c*-axis with the B_1_ field.

After selecting valid angle combinations and calculating the corresponding expected integral ratio (eq 1), an orientation dependence of *I*_eq_/*I*_ax_ for a single-domain crystal can be obtained (S3). The integral ratio primarily provides qualitative insight into the angle γ between the predominant magnetization vector of axial bromides and the B_1_ field, i.e., between the crystallographic *c*-axis and the B_1_ field. Meanwhile, the changes in angles α and β cannot be tracked. Larger ratios indicate that γ is closer to 0° or 180°, pointing to the alignment of the *c*-axis with the B_1_ field (Figure 2c). In real samples, however, multiple domains with different orientations contribute to the observed integrals of the axial and equatorial bromide signals. Each domain follows the aforementioned sinusoidal dependence, scaled by the relative size and number of coherently aligned domains. Consequently, the following generalized expression more accurately reflects the behavior of real samples with multiple domains:

$$\frac{I_{\mathrm{eq}}}{I_{\mathrm{ax}}}=\frac{y_{\mathrm{eq}}+A_{\mathrm{eq}}\cdot \sin(x+d_{\mathrm{eq}})}{y_{\mathrm{ax}}+A_{\mathrm{ax}}\cdot \sin(x+d_{\mathrm{ax}})}$$

To validate this theoretical framework for determining the crystallographic orientation of large crystals, a highly crystalline reference sample (S1) was characterized by using Laue diffractometry and NQR spectroscopy. NQR signals of both frequencies were acquired as a function of the orientation with respect to B_1_ (S1: Modified NQR setup; Figures S1 and S2), and the integral ratio was obtained (Figure 3a). The angle between the sample and the coil was controlled by changing a 3D-printed holder. The largest experimentally obtained integral ratio was compared to the proposed theoretical model.

**Figure 3:**
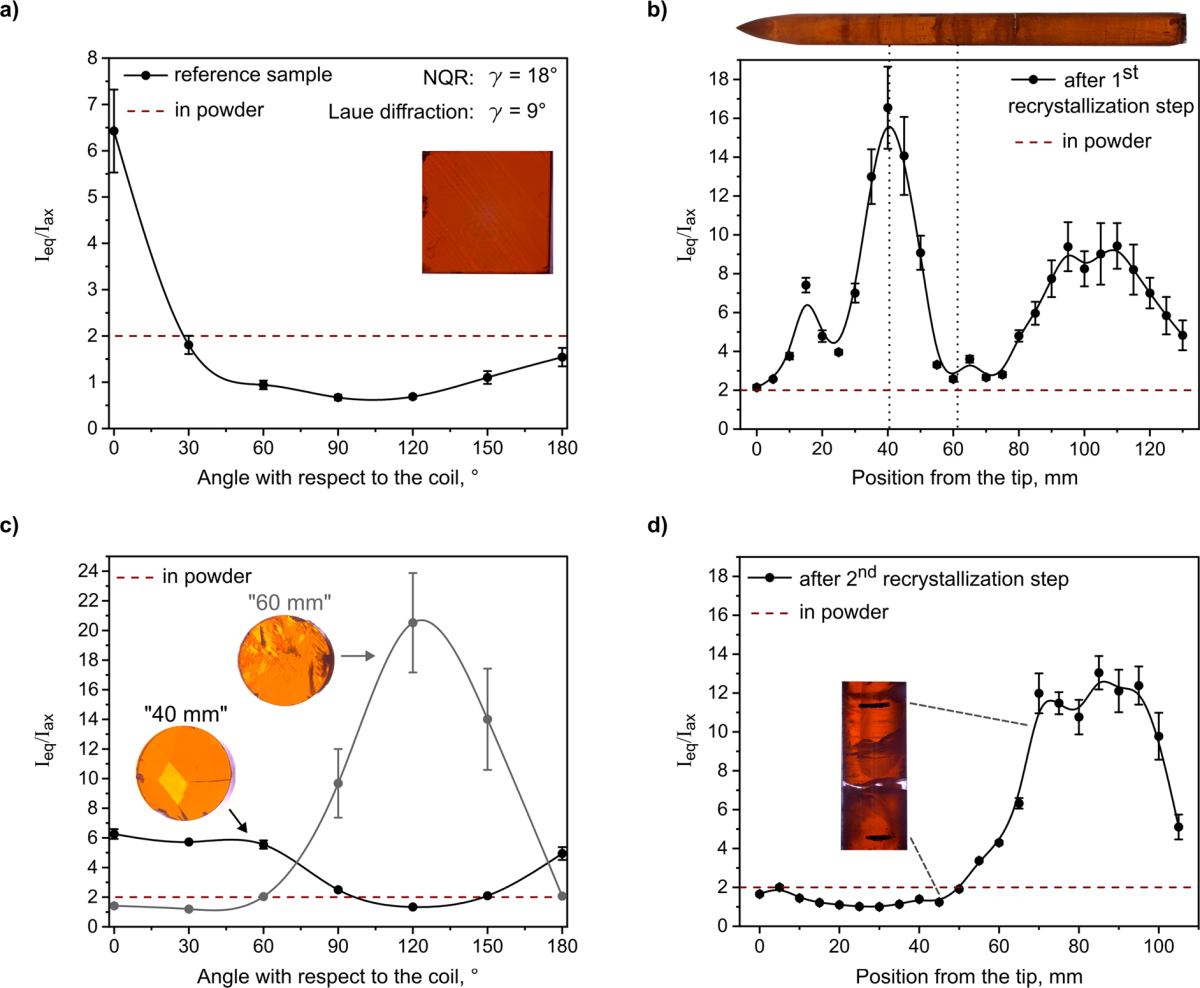
(a) Integral ratio dependence on the angle between the reference sample and the B_1_ field vector. The angle with respect to the coil is defined as the angle between the plane of the sample and the coil. Inset: image of the reference sample under polarized light. (b) Spatially resolved integral ratio of the ingot following the first recrystallization step with its image under polarized light. (c) Integral ratio dependence on the angle between disk samples and the B_1_ field vector. Inset: images of disks under polarized light. (d) Spatially resolved integral ratio of the ingot following the second recrystallization step. Inset: image under polarized light of the highlighted region. In all panels, data are shown as discrete measured points with a continuous line as a guide for the eye.

After taking into account the angle of sample with respect to the coil where the highest integral ratio was obtained (0° in this case) and under the assumption of mostly coherently aligned crystalline domains within the sample, the angle between the B_1_ field and crystallographic *c*-direction, or γ, can be estimated (Figure S5) to be 18°. Meanwhile, the crystal needed to be rotated by about 9° to align the *c*-axis with the incoming X-ray beam in a Laue diffractometer (S1: Laue diffraction). Evidently, the two methods are in reasonable agreement, rendering NQR spectroscopy a viable option to assess the alignment of CsPbBr_3_ ingots while having the advantage of analyzing the sample as a whole rather than a specific spot on a surface. In the case where the *c*-axis of the disk remains perpendicular to the B_1_ field upon rotation, the disk may also be rotated around an orthogonal axis to obtain information about orientation (Figure S6).

Using the same approach, a spatially resolved measurement (S1) was conducted on a CsPbBr_3_ ingot, following the first recrystallization step. The data was acquired in 5 mm (S4) increments along the whole ingot at both frequencies. The obtained integral ratios (Figure 3b) show fluctuations along the ingot. This indicates changes in the growth direction of the crystalline domains along the melt-grown CsPbBr_3_ ingot. At the same time, the dampened nature of the ratio indicates the presence of multiple domains that are not coherently aligned.

This is additionally confirmed by optical microscopy under polarized light (Figure 3c inset; Figure S8) of typical samples used for hard radiation detection studies. These samples were cut from two distinctly different regions of the ingot, with the highest (at 40 mm) and lowest signal integral ratio (at 60 mm) and had a thickness of 3.5 mm. Orientation-dependent NQR of the two disks showed a clear dependence of the integral ratio on the alignment with B_1_ (Figure 3c).

The estimated alignment differs by only 10° between the ingot and disk samples, indicating that the crystal orientation was preserved after cutting and polishing with the difference in absolute values resulting from the high sensitivity of the integral ratio to minor tilts. The angle for the “60 mm” disk between the growth direction and the crystallographic c-direction is estimated to be 65°, under the assumption that it contains mostly coherently aligned domains. At the same time, the 40 mm sample shows no dependence on the angle for the first 60° of rotation. Such a behavior is characteristic of the presence of multiple crystalline domains (Figure 3c inset), resulting in averaging.

Changes in the crystallographic orientation of the domains along the ingot were observed even after the second and third recrystallizations. With each recrystallization step, the crystalline domains became more homogeneously aligned, undergoing only a single reorientation each (Figure 3d, 50–70 mm; Figure S9, 60–75 mm). Notably, no apparent change in the domain orientation is visible under polarized light in these regions.

The unique power of NQR spectroscopy in assessing the crystallographic orientation of CsPbBr_3_ crystals is thus evidenced. The ability to map the crystal orientation along the ingot after growth enables the selection of the region with the best alignment of the crystal domains to tackle open questions, such as possible anisotropic charge-transport properties. Yet, without information on the crystallinity of the sample, a dampened integral ratio might cause a misinterpretation of the obtained results. To gain insights into the crystallinity of the ingots, we turn to the next spectroscopic descriptor: the linewidth.

The environment of nuclei near the crystal surface naturally differs from that of the bulk, yielding a distribution of local EFGs. The same holds for other crystal inhomogeneities, such as impurities or defects.^24,41^ The resulting variation causes inhomogeneous broadening of the NQR signal, linking the linewidth directly with sample quality. To estimate the surface effect on the linewidth, a series of DFT calculations were performed on CsPbBr_3_ clusters of three sizes (3 × 3 × 3, 4 × 4 × 4, and 5 × 5 × 5 [PbBr_6_] octahedra) based on the average bulk structure. Such an approach has previously and successfully been used to predict the NQR signatures of the respective 2D materials.^42^ Values of the quadrupolar coupling and asymmetry parameters were extracted for each ^79^Br atom in the clusters to obtain NQR frequencies as a function of the distance from the surface (Figure 4a). The calculations revealed that the two outermost layers in the cluster span a frequency range of 9 MHz and are lower in frequency. Due to the substantial overlap between the frequency distributions of the axial and equatorial bromides in the two outermost layers (Figure S10), these sites were treated as a single group in the analysis. By the third layer, the frequency distribution within the layer narrows. Yet even the fourth and fifth layers are distinct from one another in mean frequency, and they span a frequency range of 350 kHz, still causing significant inhomogeneous broadening (Figure 4a inset). Although the absolute calculated frequencies deviate from the experimental values, they nonetheless capture the trend of increasing the frequency distribution of bromides closer to the surface, which explains the signal broadening observed in smaller crystallite cores.

**Figure 4:**
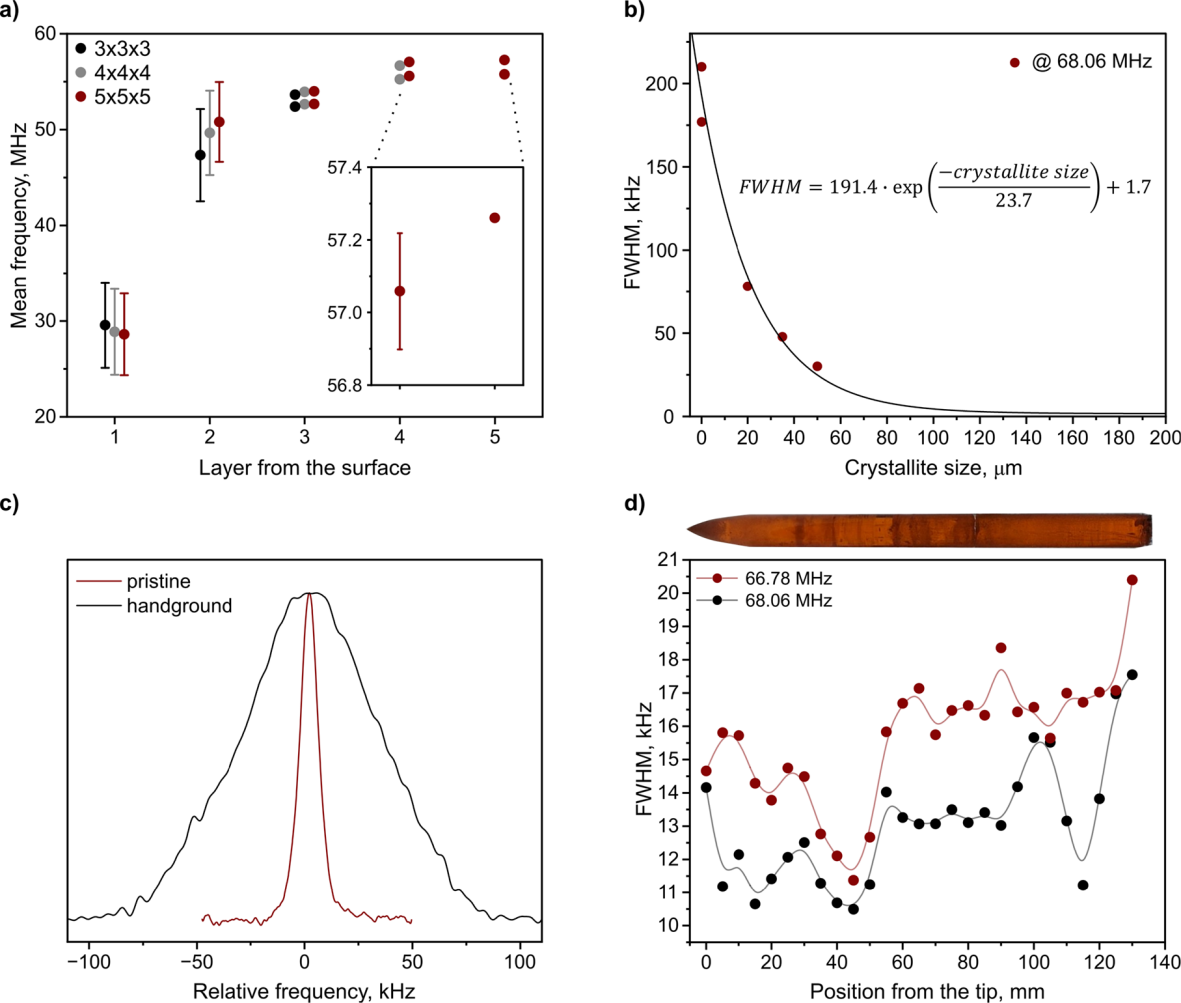
(a) Calculated ^79^Br mean frequencies for individual bromide layers, with the surface layer defined as the first, in three different sizes of CsPbBr_3_ clusters, illustrating the inhomogeneous broadening of the signal. When the frequency distribution within a layer exceeded the 1.5 MHz separation observed experimentally between axial and equatorial bromides—due to substantial overlap of the frequency distributions of the two bromide types—the frequencies were grouped together. (b) Size-dependent FWHM of the high-frequency signal corresponding to axial bromides. (c) Comparison between ^79^Br NQR spectra of pristine CsPbBr_3_ crystal used to obtain different-sized powders and handground CsPbBr_3_ powder with sizes of crystallites <20 μm. (d) Spatially resolved FWHM of the ingot following the first recrystallization step, with its image under polarized light showing discrete measured points with a continuous line as a guide for the eye. Error bars are smaller than the markers used in the plot.

We can therefore confirm that the effect of the surface reaches deep into the crystal. To experimentally highlight the influence of crystallinity on the linewidth, a series of size-dependent measurements on the high-frequency signal were conducted (Figure S11). Samples of different sizes were obtained by hand grinding a Bridgman-grown ingot and passing the powder through a sieve tower. To achieve even smaller sizes, CsPbBr_3_ nanocrystals were synthesized (S1). Significant peak broadening was observed, which decreased with an increase in crystallite size (Figure 4b). The observed peak broadening as a function of size can be described by an exponential decay, approaching a plateau of 1.7 kHz at a size of 150 μm. To isolate the inhomogeneous contribution to the linewidth, we measured the homogeneous linewidth of CsPbBr_3_, yielding the same value of 1.7 kHz for the high-frequency signal. The FWHM difference between the pristine crystal used to obtain different-sized powders and polycrystalline powder (<20 μm domains) reaches 70 kHz (Figure 4c) in line with previous reports.^29^ Given that the surface fraction for a 20 μm cubic domain is far below 1%, the observed broadening cannot be solely attributed to the outermost surface layer, further confirming the large structural probing range.

To assess the crystallinity of the melt-grown CsPbBr_3_ ingots, a spatially resolved measurement with a step size of 5 mm was performed. The first notable observation is that the FWHM of the axial bromides’ signal is systematically narrower compared to the equatorial along the whole ingot (Figure 4d). Considering that this trend was also observed for CsPbBr_3_ powder (Figure 1b), we attribute it to the low-frequency signal consisting of two closely overlapping peaks of the equatorial bromides. Second, the measurement revealed a nonuniform peak width along the ingot spanning a frequency range from 20 to 10 kHz. Given the homogeneous linewidth of 1.7 kHz, we conclude that the melt-grown CsPbBr_3_ is polycrystalline. For both signals, the peak width is broader toward the top of the ingot, likely due to impurity accumulation and possibly a more defective structure, as expected from Bridgman growth. However, fluctuations of the FWHM in the middle part of the ingot and especially near the tip, where the material is expected to be of the highest purity, suggest the distribution of crystallite domain sizes. In this case, the tip region experiences a supercooling effect that can reach 20–30 °C,^43^ causing instant melt crystallization and formation of a polycrystalline part. Directional crystallization begins after supercooled crystallization.

To track the evolution of crystal quality across several recrystallization steps, which are expected to improve crystal quality, the same ingot was remeasured after the second and third recrystallization steps (Figure 5; Figure S12). First, the distribution of the FWHM becomes narrower after each step, signifying that the material homogeneity along the crystal indeed improves (Figure 5). Second, the average FWHM of the peaks decreases, indicating bigger average domains. The crystalline domains are estimated to be 70–80 μm based on our “NQR sizing curve” (Figure 4b). These findings confirm that a multistep purification procedure, consisting of impurity removal and recrystallization, improves the sample’s crystallinity. Moreover, no significant changes in the crystallinity were observed after cutting the ingots and polishing the disks (Figure S13). Overall, we established that NQR spectroscopy is a powerful tool for quality control of melt-grown LHP crystals.

**Figure 5:**
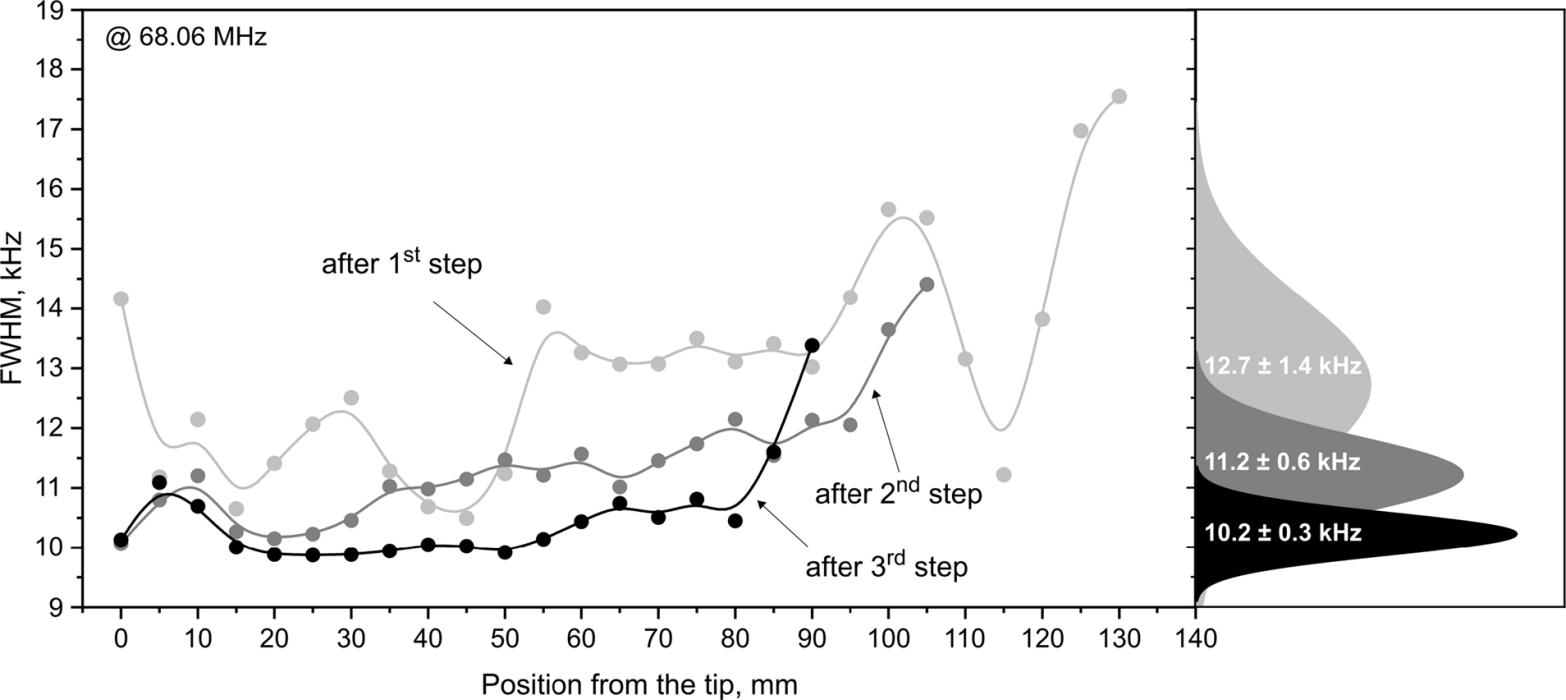
Spatially resolved FWHM (high frequency, axial bromides) of the ingot following the first, second, and third recrystallization steps along with the distribution of corresponding FWHM values plotted on the right. All data points represent discrete measurements with a continuous line as a guide for the eye. Error bars are smaller than the markers used in the plot.

## Conclusions/Outlook

In this work, we establish NQR spectroscopy as a noninvasive method for assessing the quality of melt-grown CsPbBr_3_ ingots in spatially resolved manner. This enables the precise identification of high-quality crystal regions without the need for destructive analysis or full device fabrication. By directly probing the as-grown ingot in its sealed ampule, we establish a practical approach to correlate spectroscopic descriptors, specifically, linewidth and integral ratios, with fundamental structural characteristics such as the crystallinity and crystallographic alignment. Spatial mapping across the ingot reveals not only regions of enhanced crystallinity but also reorientations in crystal-domain alignment preserved after mechanical processing. Moreover, the significant improvements observed in crystal quality (crystallinity) and domain alignment in the ingots, achieved through multistep directional crystallization, indicate a promising way for optimizing the crystal growth process to obtain high-quality detector-grade material. Furthermore, the implementation of orthogonal field configurations could enhance future generations of NQR probes, enabling multidimensional mapping of spectroscopic descriptors and thus providing deeper insights into the spatial heterogeneity of crystalline materials. We anticipate integrating NQR-based diagnostics into existing workflows in both in-line and in-situ manners, e.g., during the crystal growth or processing of the material, as well as in-operando, e.g., during the testing and operation of the final electronic devices.

## Experimental Section

### Bridgman-Stockbarger Crystal Growth of CsPbBr_3_

A clean quartz ampule with an inner diameter of 10 mm was loaded with stoichiometric amounts of CsBr (>99.999%, Sigma-Aldrich) and PbBr_2_ (prepared in-house). The ampule was evacuated to 10^–3^ mbar and subsequently flame-sealed. Afterward, it was placed in a muffle furnace at 700 °C for 10 h to homogenize and synthesize the perovskite, followed by natural cooling to room temperature. The ampule containing the bright-orange polycrystalline CsPbBr_3_ was positioned in the hot zone (>600 °C) of a Bridgman furnace. The cold zone was set to 450 °C, which provided a temperature gradient in the furnace of 20–30 °C/cm. After the material was fully molten, the ampule was slowly moved (0.3–1 mm/h) toward the cold zone, allowing the single crystal to grow. During the multistep recrystallization, this procedure was repeated several times. After each step, the top section (1–2 cm) of the grown ingot was discarded by chipping the crystal to avoid the introduction of impurities by processing steps such as cutting. The remaining crystal was then loaded into another ampule, and the growth process was repeated.

### NQR Measurements

Measurements were performed on a modified commercial static broadband 400 MHz NMR probe containing a single variable vacuum tube capacitor (2–32 pF) with a 3D-printed attachment (Figure S1a). The probe was connected to a Bruker Avance III HD console. All spectra were acquired as an echo to remove probe ringing, starting the acquisition at the top of the echo.

### Modified NQR Setup

The 3D printable attachment contains an excitation/detection inductor made from coated copper wire, a resistor (10 Ohm), and a match coil (Figure S1a) to match the 50 Ohm impedance of the spectrometer. With this, we can achieve an attenuation of 60 dB with minimal resonant ringing across the required frequency range (60–70 MHz). The excitation/detection inductor is shorter (2 mm) than the ingot (up to 15 cm) and therefore enables spatially resolved measurements by moving the ingot relative to the coil. To facilitate such measurements in a timely and controlled manner, the setup was further expanded. The addition of a frame, a stepper motor (including a driver), a linear translational stage, and a control unit enabled control of the relative position of the ingot with respect to the probe. Using an Arduino microcontroller running custom code, the spectrometer triggered the stepper motor via the pulse program’s trigger function. To avoid interference coming from the stepper motor, its power was automatically cut during each individual measurement (Figure S1a,b).

### DFT Calculations

DFT calculations were performed using the Amsterdam Modeling Suite run on the Euler cluster. Generalized gradient approximation (GGA) with the Perdew–Burke–Ernzerhof exchange-correlation functional (PBE) was used for single-point calculations, including EFG tensor calculations. The calculation was performed using a double-ζ basis set without a polarization function (DZ), due to the size of the system. The structures of three clusters of different sizes were obtained by expanding the average bulk structure of CsPbBr_3_, based on previously published structural data.^38^

When the distribution of bromide frequencies at each site exceeded the 1.5 MHz difference observed between axial and equatorial bromides in the experiment, the signals were treated as a single group in the calculations.

## Supplementary Material



## Notes

Detailed description of the experimental procedures, measurement parameters, NQR setup, and additional figures (PDF)
