# Supplementary material for: Noninvasive Quality Assessment of Melt-Grown Cesium Lead Bromide Perovskite by Nuclear Quadrupole Resonance Spectroscopy
Source: Chem Mater. 2026 Jan 8;38(2):715–23. doi: 10.1021/acs.chemmater.5c02047 (PMC12856995; doi:10.1021/acs.chemmater.5c02047)
Supplement: Supplementary file 1 [file cm5c02047_si_001.pdf]

Supporting information for

Noninvasive Quality Assessment of Melt-Grown Cesium Lead Bromide Perovskite by Nuclear Quadrupole Resonance Spectroscopy

Lidiia Dubenska[‡,1,2], Sebastian Sabisch[‡,1,2], Andrii Kanak[1,2], Martin Kotyrba[1,2], Maksym V. Kovalenko[*,1,2]

[1] ETH Zürich, Department of Chemistry and Applied Biosciences, Vladimir-Prelog-Weg 1-5, Zürich CH-8093, Switzerland

[2] Empa-Swiss Federal Laboratories for Materials Science and Technology, Überlandstrasse 129, Dübendorf CH-8600, Switzerland

Email: mvkovalenko@ethz.ch

S1. Additional experimental details

Reference sample for orientation measurement

The reference sample was prepared from a high-quality, melt-grown $CsPbBr_3$ ingot, selecting an especially transparent region. The ingot was grown with two recrystallization steps using the same procedure as those used in the study, and the final dimensions were 5 mm x 5.2 mm x 1.6 mm. Orientation-dependent NQR measurements were performed first, revealing the high crystallinity of the sample through consistently narrow linewidth, followed by Laue diffraction to confirm the crystallographic orientation.

Size selected samples $CsPbBr_3$

Three powder samples with crystallite sizes below 20 μm, between 20-50 μm and above 50 μm were obtained by hand-grinding of a Bridgman-grown ingot. A sieve tower was used for size selection. To achieve even smaller sizes, nanocrystals with crystalline core sizes of 25 nm and 35 nm were synthesized according to Morad et al. and Akkerman et al.[1,2]

NQR measurements

All spectra were acquired as an echo, starting the acquisition at the top of the echo with the following parameters:

Carrier frequency	66.797 MHz / 68.058 MHz
Spectral width	100 kHz
Echo delay	4.88 μs
Recycling delay	0.1 s
π/2 pulse length	20 μs
Input power	300 W
Number of scans	Depending on the sample, typically 8192
Acquisition length (number of points)	1024
Dwell time	5 μs

The size-dependent measurements were performed on a commercial broad-banded 400 MHz 3.2 mm HX probe due to the wider achievable excitation bandwidth. In the case of NCs, the width of the signal surpassed the excitation and detection bandwidth of the probe. Therefore, a variable offset cumulative spectra (VOCS) acquisition approach was employed.

The homogenous linewidth was measured using a Hahnecho pulse sequence with a variable echo delay. The signal is refocused after the initial excitation and the intensity is analysed as a function of the delay between excitation and refocusing pulse. A Fourier transformation of the signal decay yields the homogenous linewidth.

Modified NQR setup

At the start of the experiment, the control unit was first used to adjust the initial position of the ingot and the desired step length for spatially resolved measurements was set in the Arduino code. Afterwards the spectrometer was programmed to send a pulse to the microcontroller unit which moved the ingot by the desired length before starting a next measurement. The ingot was mounted to a flexible wire attached to an arm above the probe. A calibrated stepper motor lowers the ingot in precise increments to control its spatial position while keeping its orientation fixed (Figure S1). This allowed us to record a pseudo two-dimensional spectrum where the indirect dimension is the relative position of the ingot with respect to the coil. The total experiment time for such a measurement is 1-5 hours depending on the length of the ingot and the desired number of scans per slice. The orientation-dependent measurements were performed only on cuts from the grown ingots with an angular resolution of 30°. The angular orientation of the sample with respect to the coil was controlled by 3D printing several holders with rotated sample spaces (Figure S2). Printing of each holder only takes about 20 minutes, and the sample space can be adjusted to fit to the exact dimensions of the sample. Holders were mounted to a linear stage (Figure S2) allowing for precise positioning of the sample withing the coil. Technically the size of the rotational steps can be adjusted to the need of user limited only by the precision of modern FDM printers.

a)

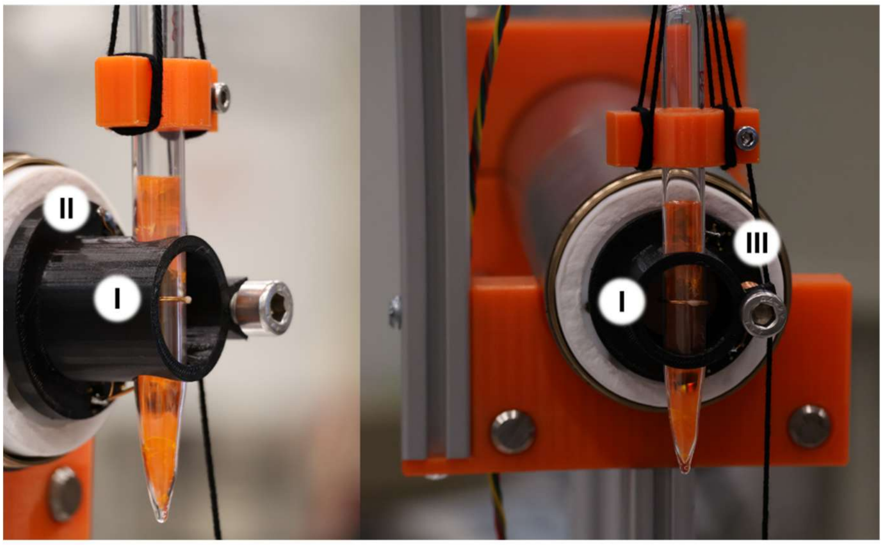

b)

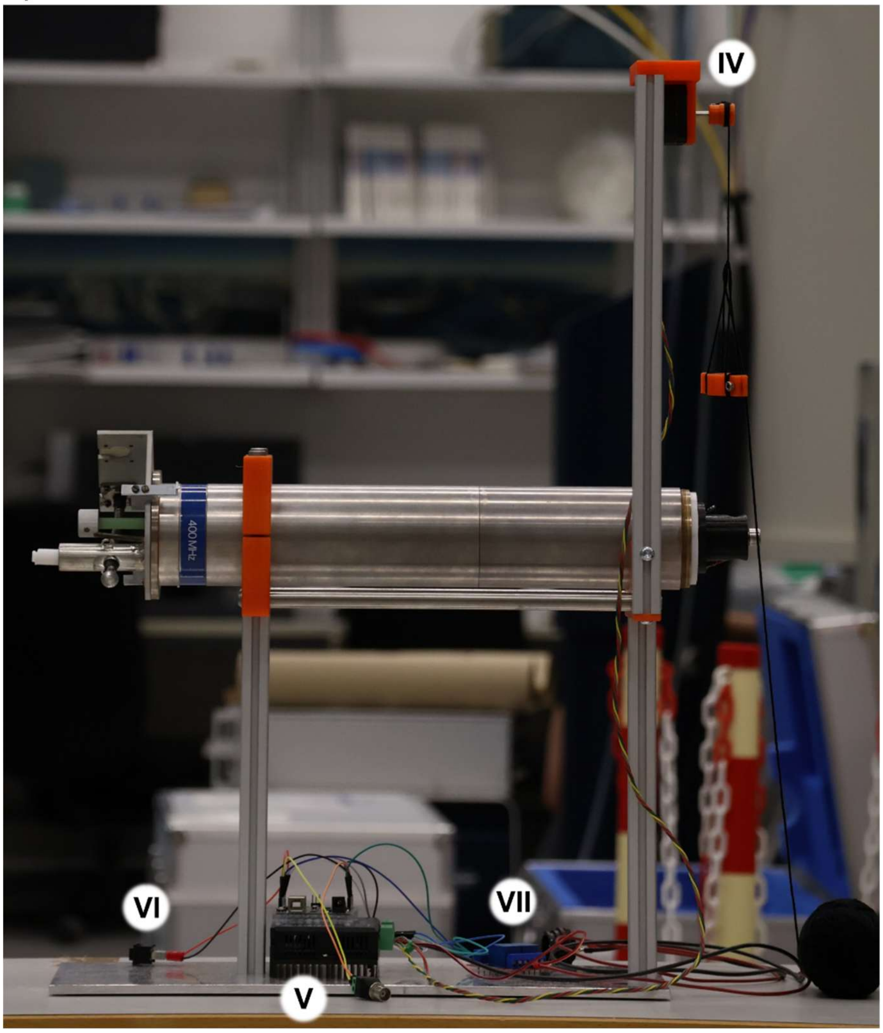

Figure S1. a) 3D printed attachment connected to a commercial broad-banded 400 MHz probe containing (I) an excitation/detection inductor, (II) a resistor, and (III) a variable inductor to match the 50 Ohm impedance of the 1000 W amplifier. **b)** Setup for NQR measurement of the ingots which includes a frame, (IV) stepper-motor (including the driver), (V) a control unit, (VI) end-switches, and (VII) a switch that allows the spectrometer to turn of the power of the stepper motor during the measurement.

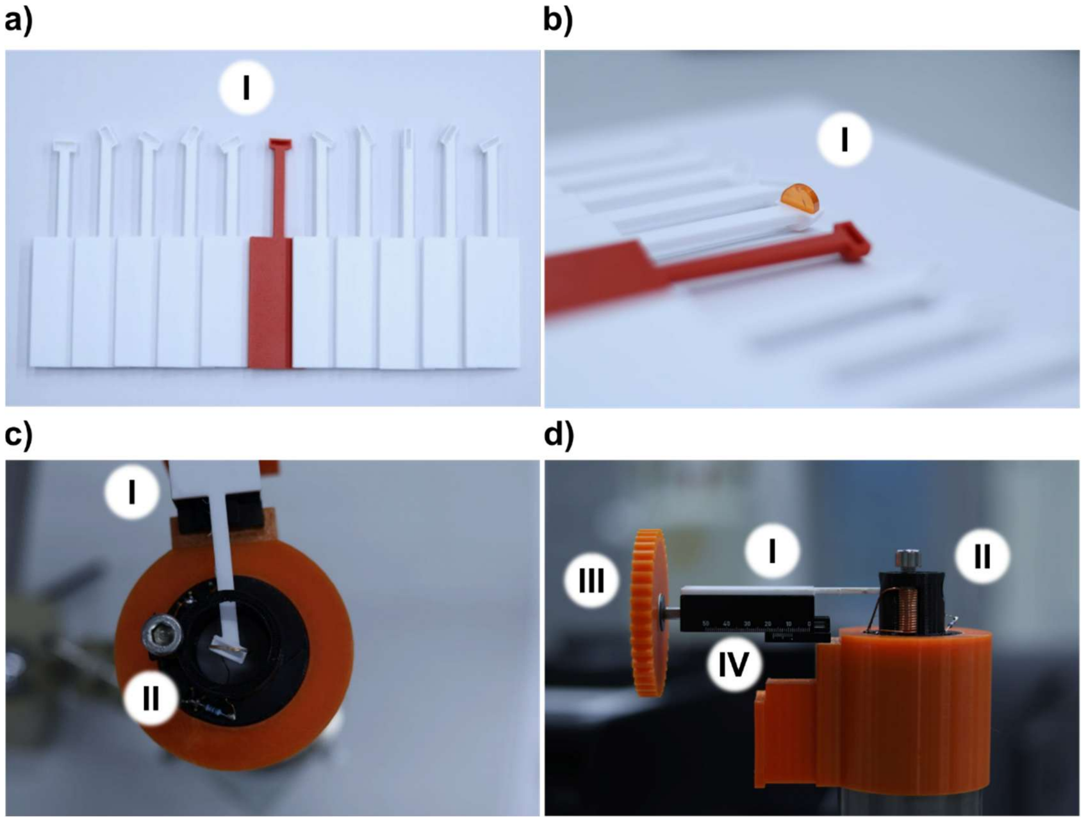

Figure S2. a) Library of (I) 3D printed holders for orientation dependent NQR measurements of disk-shaped samples. **b)** (I) 3D printed holder for orientation dependent NQR measurement with a disk-shaped sample mounted in the sample space. **c)** Top view of (I) the 3D-printed holder used for orientation-dependent measurements, mounted within (II)the measurement setup. **d)** Side view of (I) the 3D-printed holder used for orientation-dependent NQR measurements, mounted within (II) the measurement setup. The (III) gear mechanism can be driven by a stepper motor positioned beneath (IV) the linear stage, enabling control of the sample position during the measurement.

Laue diffraction

Laue data was acquired on ARL EQUINOX LAUE X-Ray diffractometer equipped with Mo source. Laue diffraction data was processed using the CLIP software. The sample was rotated until the c-axis of the crystal was aligned with the incoming X-ray beam. We note that the exact result of the measurement depends on the specific spot measured on the crystal, most likely due to the presence of twin domains, which is also supported by optical microscopy under polarized light (Figure 3a, inset). The crystal needed to be rotated by about 9° to align the X-ray beam with the crystallographic c-axis, we estimate the error of such a measurement to be about 5°.

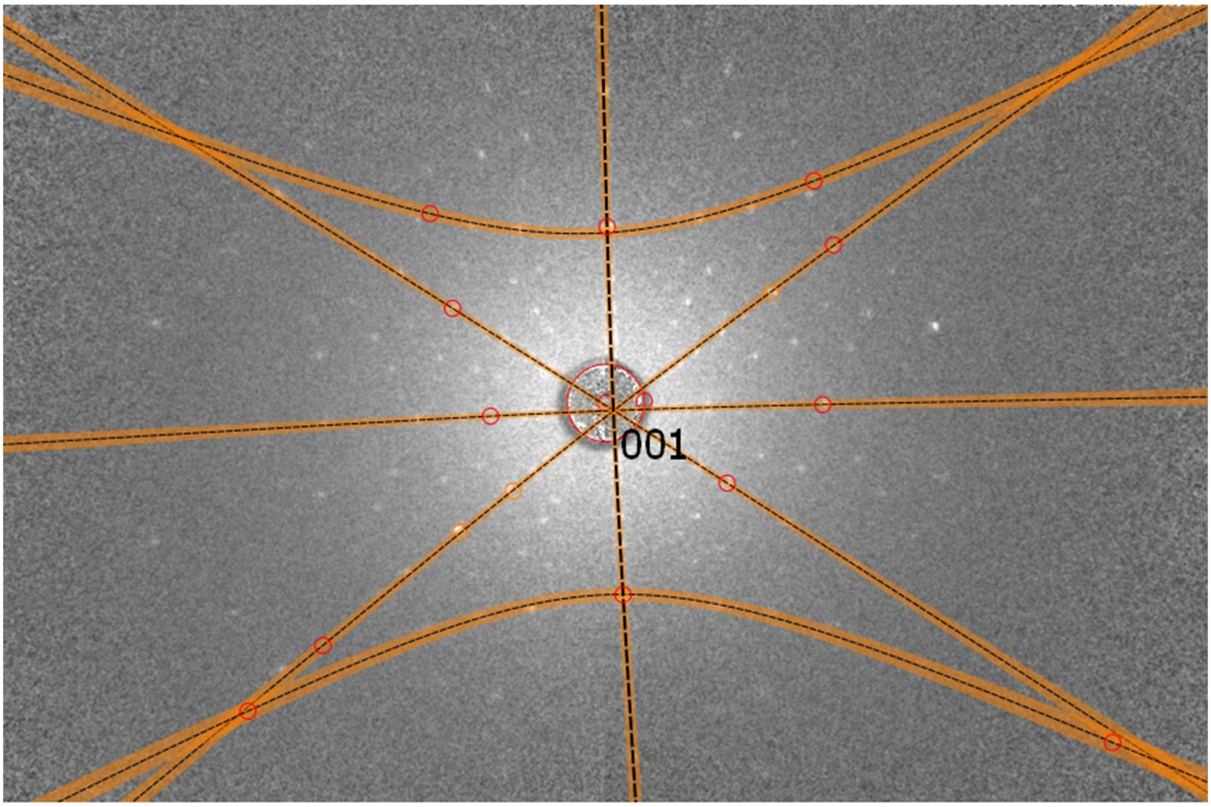

Figure S3. Laue diffraction of the reference sample shows that the X-ray beam is aligned with the crystallographic c-axis. The crystal needed to be rotated by about 9°.

Table S1. Nuclear resonance properties of quadrupolar nuclei found in LHPs.

Nucleus	Spin	Quadrupole moment Q (e·fm-2)	Natural abundance, %
^{35}Cl	3/2	–8.165	75.76
^{37}Cl	3/2	–6.435	24.24
^{79}Br	3/2	30.5	50.69
^{81}Br	3/2	25.4	49.31
^{127}I	5/2	–71	100

S2. NQR spectroscopy – peak frequency

The transition frequency detected by NQR spectroscopy is determined by quadrupolar interaction – the coupling between the quadrupole moment (Q), which is the result of an asymmetrical charge distribution in the nucleus, and the intrinsic electric field gradient (EFG) around the nucleus. The EFG is determined by the deviation of the charge density around the nucleus from spherical symmetry. It is represented by a traceless second-rank tensor that can be described by the principal components (V_{11}, V_{22}, V_{33}) with the convention that $|V_{33}| \geq |V_{22}| \geq |V_{11}|$. The magnitude of the quadrupole interaction is characterized by the quadrupolar coupling constant, C_Q (Eq. 3), where e is the fundamental charge and h is the Planck's constant.

$$C_Q = \frac{eV_{33}Q}{h}. \tag{3}$$

The transition frequency is determined by the energy difference between the states with different nuclear magnetic quantum numbers, m:

$$\nu_Q = \frac{E_{m+1} - E_m}{h}, \tag{4}$$

where the energies of the available quadrupolar states for nuclei with spin quantum number I can be computed as follows:

$$E_m = \frac{eQV_{33}[3m^2 - I(I+1)]}{4I(2I-1)}. \tag{5}$$

For I = 3/2 we arrive at the expression:

$$\nu_Q = \frac{eQV_{33}}{2h} = \frac{C_Q}{2}. \tag{6}$$

In the case of a symmetrical charge distribution around the nucleus, all principal components of the EFG tensor are equal to zero meaning that there is no quadrupolar coupling and consequently no NQR resonance.

Transition frequencies for other values of I and m can be obtained similarly. For example, for I = 5/2 the transition ±3/2 → ±5/2 will be observed at twice the frequency of the transition ±1/2 → ±3/2 if the EFG tensor is axially symmetric. However, deviations from the axial symmetry of the EFG tensor will cause the drift of the transition frequencies. The axial symmetry of the EFG tensor is described by the asymmetry parameter and is determined as:

$$\eta = \frac{V_{11} - V_{22}}{V_{33}}. \tag{7}$$

Since the EFG tensor is traceless, the value of η ranges between 0 and 1, reaching 0 in the case of axial symmetry. For I = 3/2 the expression that describes the dependence on η has the following form:

$$\nu_Q = \frac{C_Q}{2}\sqrt{1 + \frac{\eta^2}{3}}. \tag{8}$$

S3. $CsPbBr_3$ signals angle dependence – theoretical considerations

Due to the orthogonality of the three magnetization vectors present in the $CsPbBr_3$, not all combinations of α, β and γ angles (Fig. S4a) with respect to the B_1 field are possible. Projections of the B_1-vector on the coordinate system built from the orthogonal magnetization vectors are described as (Fig. S4b):

$$a_p = B_1 \cos(\alpha)\,; \quad b_p = B_1 \cos(\beta)\,; \quad c_p = B_1 \cos(\gamma).$$

At the same time, from the Pythagorean theorem (Fig. S4c):

$$x^2 + c_p^2 = B_1^2,$$

where x is the projection of B_1 on the ab-plane. Therefore (Fig. S4d) the following expressions hold:

$$x^2 = a_p^2 + b_p^2; \qquad a_p^2 + b_p^2 + c_p^2 = B_1^2.$$

$$(B_1 \cos(\alpha))^2 + (B_1 \cos(\beta))^2 + (B_1 \cos(\gamma))^2 = B_1^2;$$

$$\cos(\alpha)^2 + \cos(\beta)^2 + \cos(\gamma)^2 = 1.$$

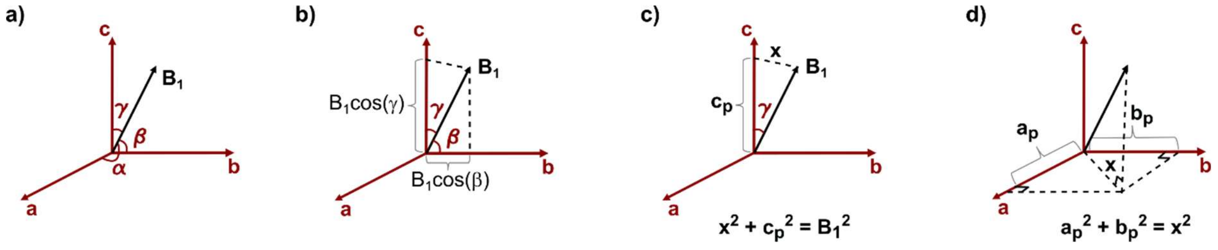

Figure S4. a)-d) Schematic description of the geometrical restrictions imposed on the possible combinations of α, β and γ by orthogonality of the a, b and c magnetization vectors that orient themselves along the highest principal value of corresponding EFG tensors.

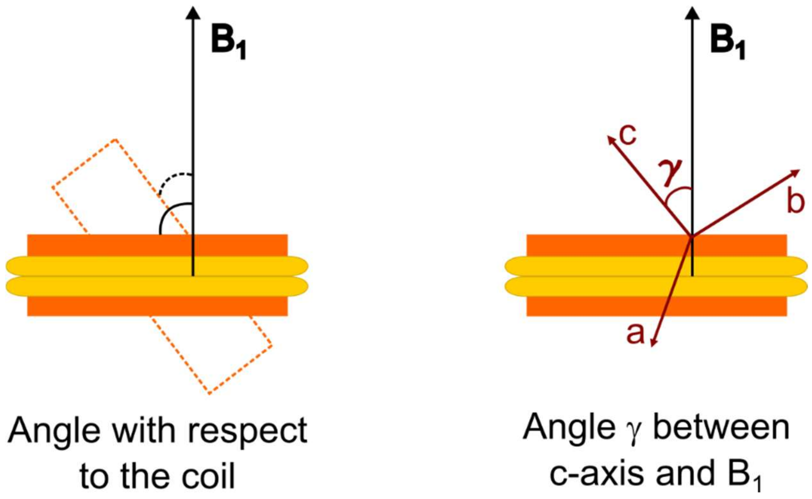

Figure S5. Schematic visualization of the definitions used for the “angle between the sample and the coil” and the angle γ.

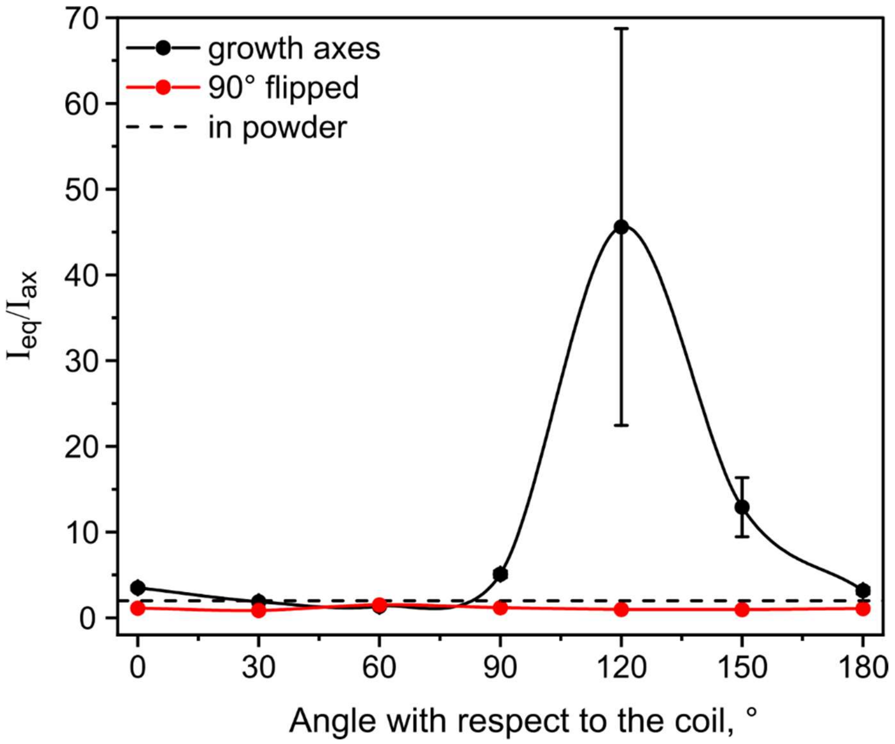

Figure S6. Integral ratio as a function of disk orientation with respect to the coil, measured for two rotation geometries: rotation around the growth axis and rotation around the axis perpendicular (90°) to the growth direction, corresponding to the typical orientation dependence reported in the paper.

$CsPbBr_3$ signals angle dependence – experimental data processing

The theoretical integral ratio and experimental integral ratio can only be qualitatively compared to assess dominant (if present) preferential orientation of crystallite domains in the sample. We consider the highest integral ratio and compare the value with the theoretical curve. Taking the angle γ obtained from that comparison and the angle of the sample with respect to the coil into account (see comment below) we estimate the alignment of domains with the growth direction.

S4. Spatial homogeneity of the generated B_1 field.

We performed a spatially resolved measurement on a disk-shaped sample with a diameter of 10 mm, scanning along two perpendicular axes of the sample's cross-section with a step size of 0.2 mm (Figure S5). We compared these experimental results with the simulated projected density of the sample (Figure S7). As shown in the figure below, the inhomogeneity of the coil broadens the shape by 1.4 mm in each direction. Based on this result we conclude that the 5 mm step size used in our spatially resolved ingot measurements is appropriate and provides sufficient spatial resolution.

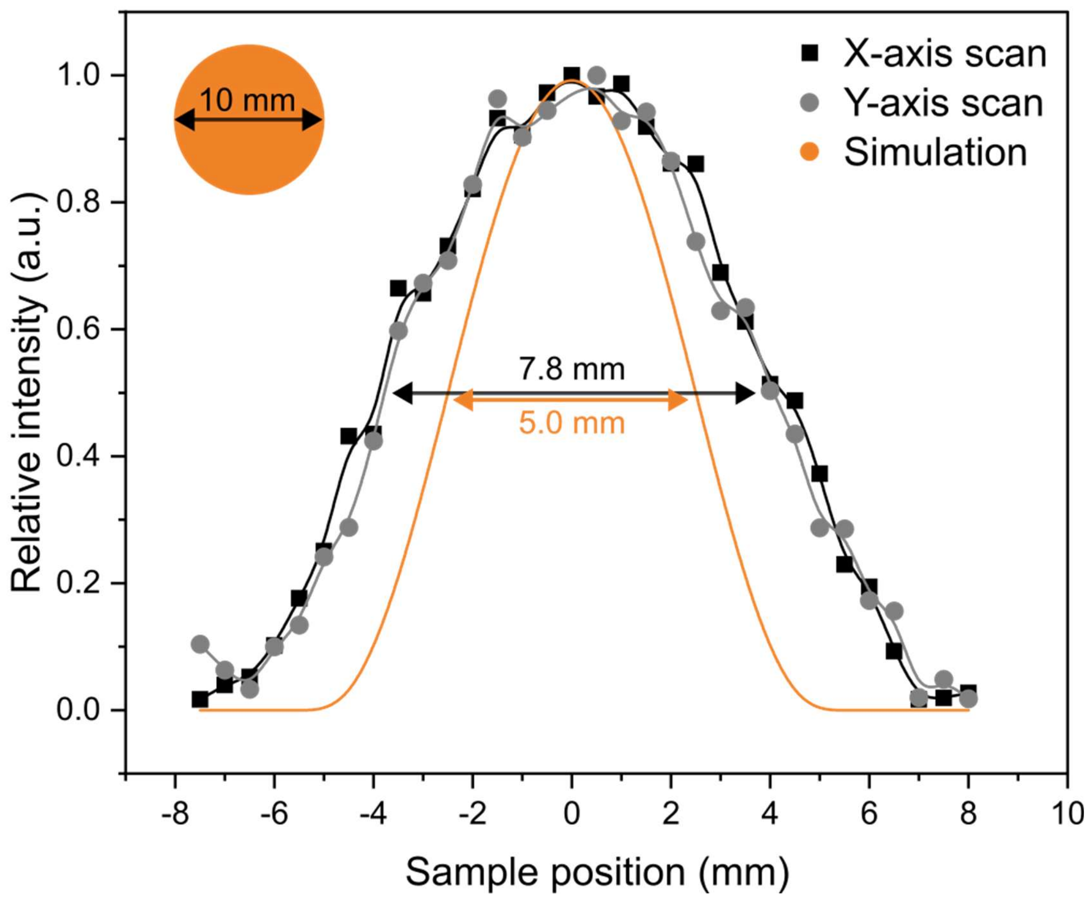

Figure S7. Comparison between spatially resolved measurements on a disk-shaped sample (10 mm diameter), scanned along two perpendicular axes of the cross-section with 0.2 mm step size, and the simulated projected signal density of the sample.

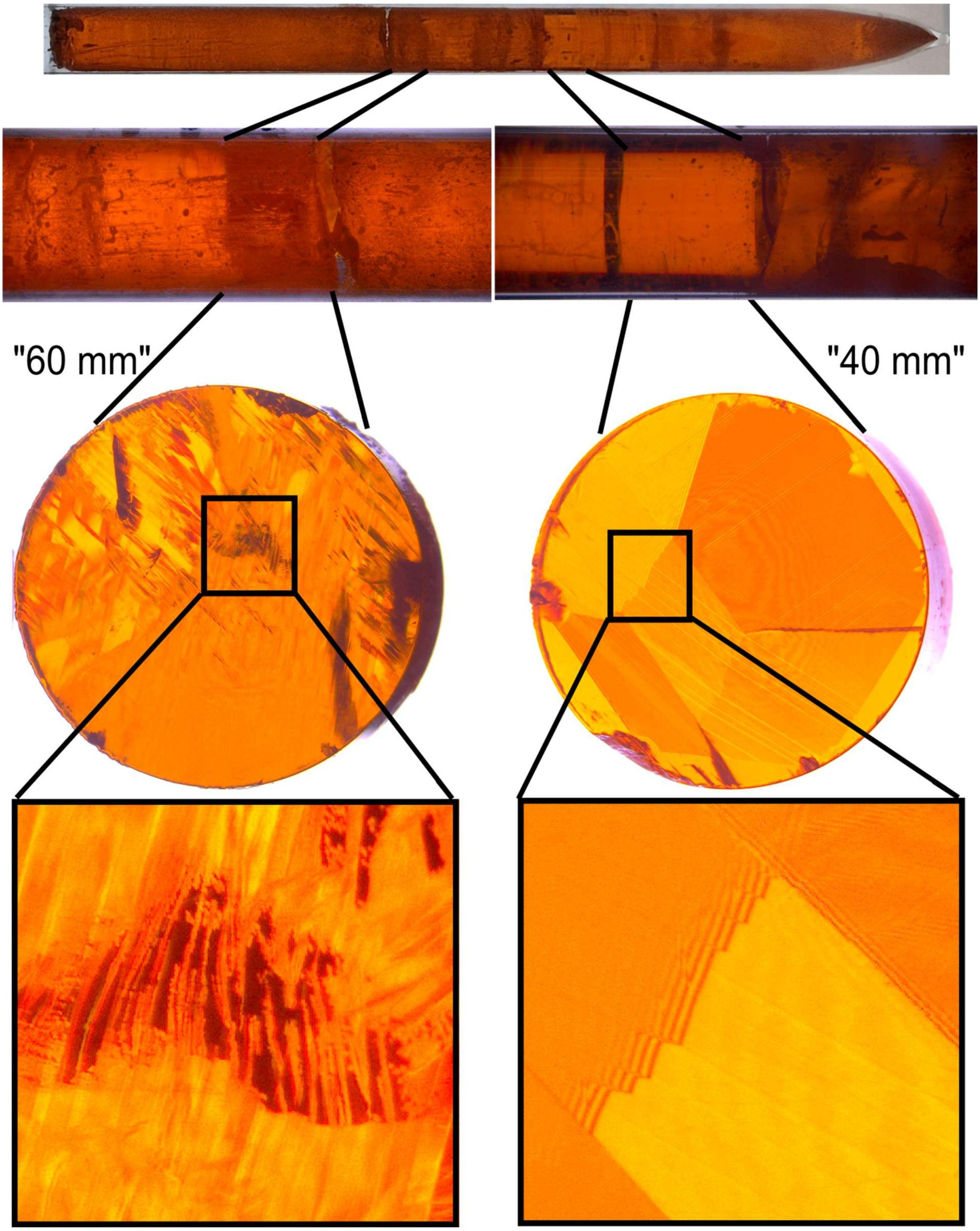

Figure S8. Images under polarized light of the $CsPbBr_3$ ingot following the first recrystallization step with corresponding disk samples typically used for hard radiation detection studies. Contrasting colors of the images indicate polycrystalline nature of the crystal with varying orientation of crystalline domains. Disk samples were cut from two distinctly different regions of the ingot, with the lowest (at 60 mm, on the left) and highest (at 40 mm, on the right) signal integral ratio I_{eq}/I_{ax} that is indicative of the preferential domain orientation. Length of the ingot: 13 cm. Disk diameter: 10 mm.

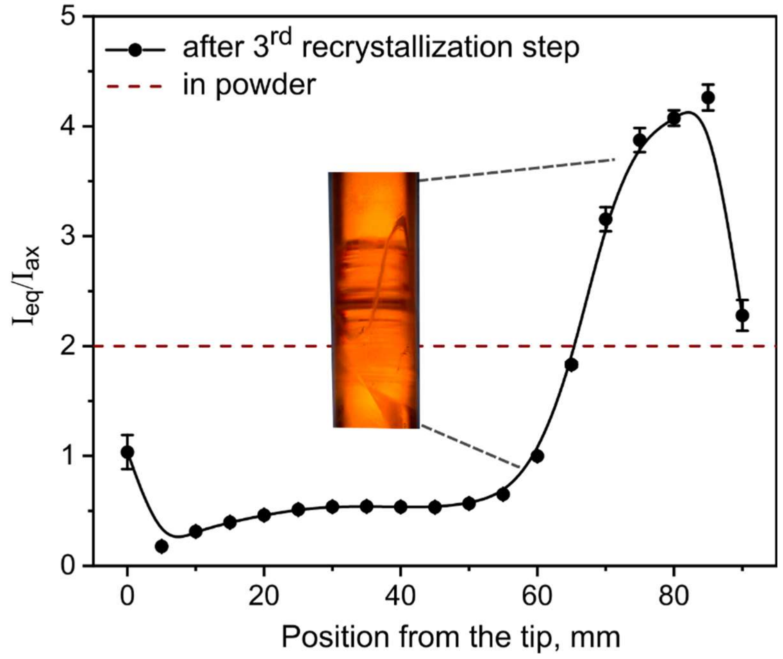

Figure S9. Spatially resolved integral ratio of the ingot following the third recrystallization step undergoing only a single reorientation of domains at 60-75 mm. Inset: image under polarized light of highlighted region with no apparent change in the domain orientation visible. All data points represent discrete measurements with continuous line as a guide for the eye.

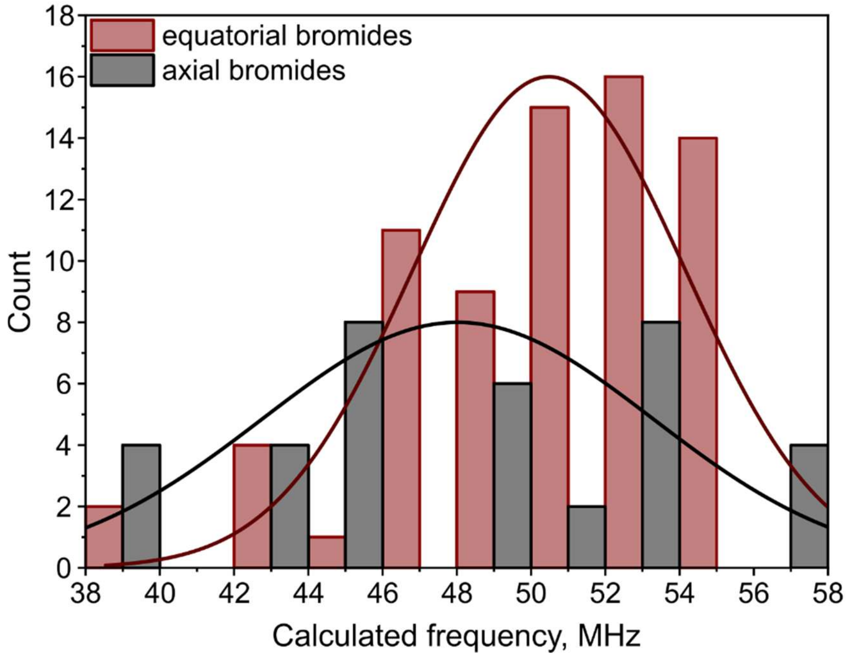

Figure S10. Distribution of calculated frequencies for axial and equatorial bromides in the second layer from the surface of a 4×4×4 [$PbBr_6$] octahedra cluster.

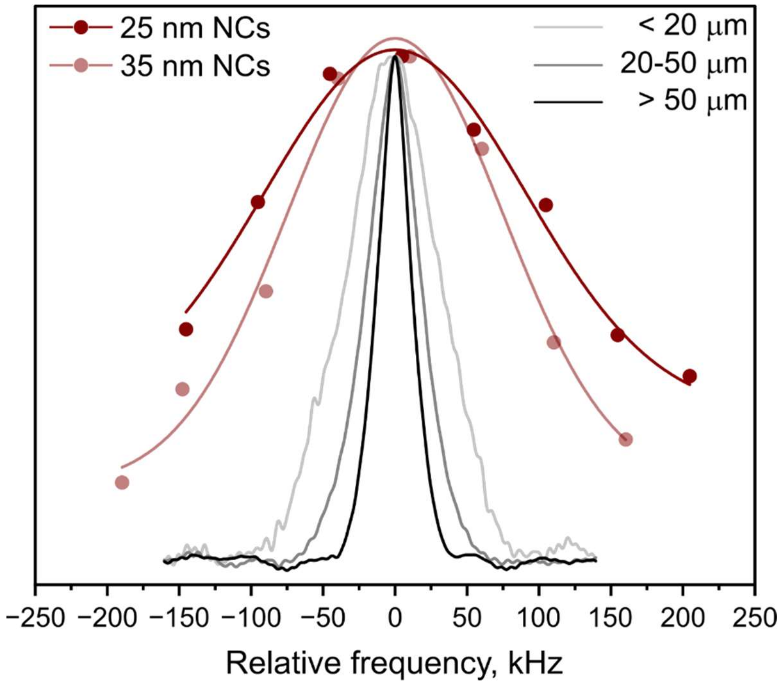

Figure S11. Normalized ^{79}Br NQR spectra of hand-ground $CsPbBr_3$ powders with sizes of crystallites < 20 μm, 20-50 μm, > 50 μm and nanocrystals with the crystalline core sizes of 25 nm and 35 nm. For the nanocrystals, spectra were acquired using an offset approach due to the linewidth exceeding the excitation bandwidth, and subsequently fitted with a Gaussian function.

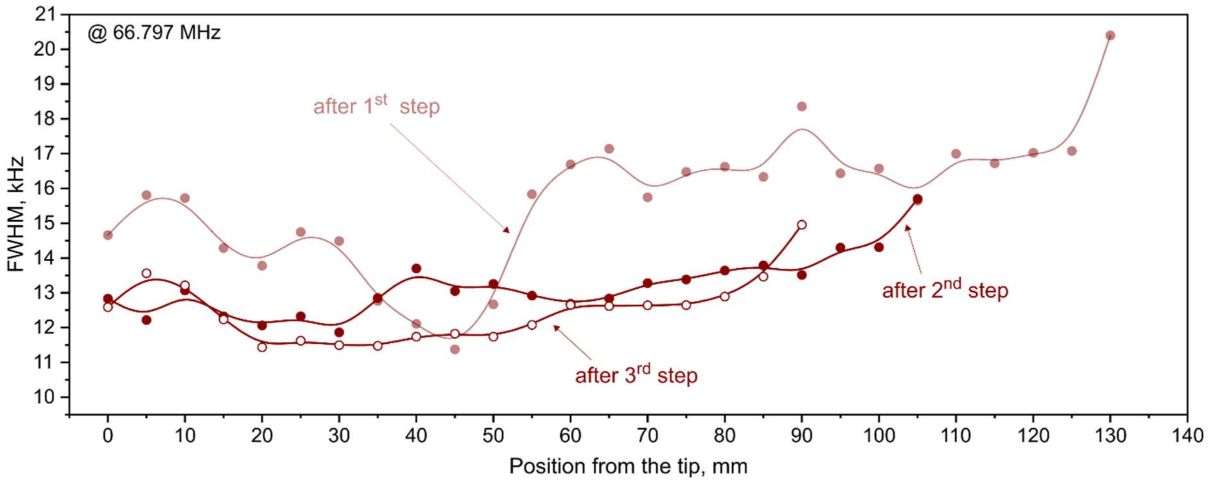

Figure S12. Spatially resolved FWHM (low frequency, equatorial bromides) of the ingot following the first, second, and third recrystallization steps showing improved average value of FWHM as well as its distribution. These findings confirm that a multiple-step purification procedure consisting of the removal of impurities and recrystallisation has a positive effect on the crystallinity of the $CsPbBr_3$ ingots.

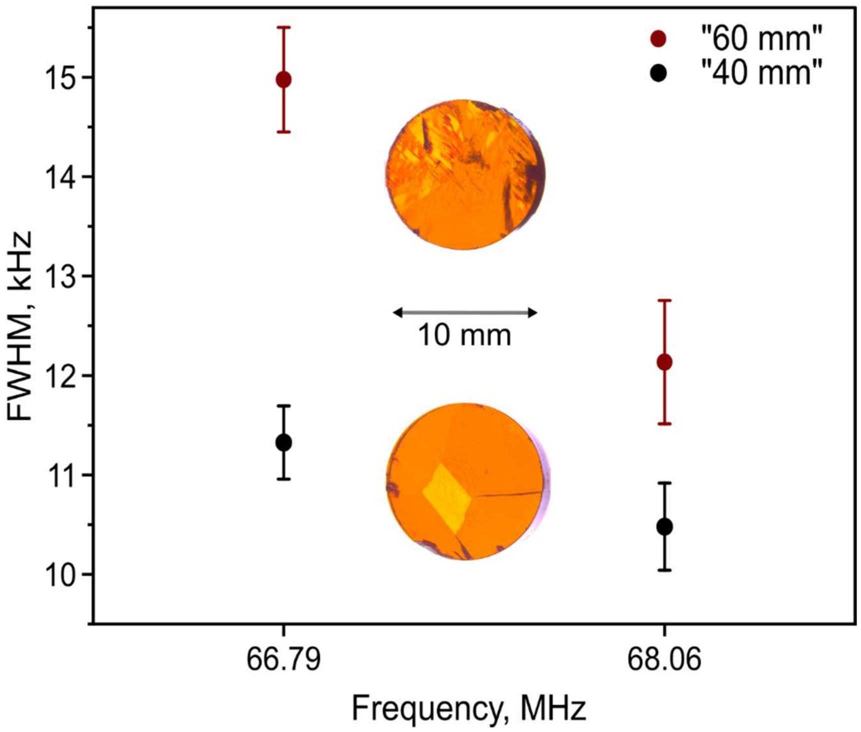

Figure S13. FWHM values after cutting of the ingot and polishing of the disk samples showing no significant change compared to the respective regions in the ingot. Disk samples were cut from two distinctly different regions of the ingot, with the highest (at 40 mm) and lowest (at 60 mm) signal integral ratio I_{eq}/I_{ax} that is indicative of the preferential domain orientation. Insets: images of the disks under polarized light.

References

(1) Morad, V.; Stelmakh, A.; Svyrydenko, M.; Feld, L. G.; Boehme, S. C.; Aebli, M.; Affolter, J.; Kaul, C. J.; Schrenker, N. J.; Bals, S.; et al. Designer phospholipid capping ligands for soft metal halide nanocrystals. *Nature* **2024**, *626* (7999), 542-548. DOI: 10.1038/s41586-023-06932-6.

(2) Akkerman, Q. A.; Nguyen, T. P. T.; Boehme, S. C.; Montanarella, F.; Dirin, D. N.; Wechsler, P.; Beiglböck, F.; Rainò, G.; Erni, R.; Katan, C.; et al. Controlling the nucleation and growth kinetics of lead halide perovskite quantum dots. *Science* **2022**, *377* (6613), 1406-1412. DOI: 10.1126/science.abq3616.
